# General practitioners believe that hypnotherapy could be a useful treatment for irritable bowel syndrome in primary care

**DOI:** 10.1186/1471-2296-5-22

**Published:** 2004-10-13

**Authors:** Stephen Cox, Simon de Lusignan, Tom Chan

**Affiliations:** 1 Gillets Surgery, Deanland Road, Balcome, West Sussex, RH17 6PH, UK; 2 Department of Community Health Sciences, St. George's Hospital Medical School, LONDON, SW17 0RE, UK; 3 Surrey and Hampshire Borders NHS Trust, Ridgewood Centre, Old Bisley Road, Camberley, Surrey, GU16 5QE, UK

## Abstract

**Background:**

Irritable bowel syndrome is a common condition in general practice. It occurs in 10 to 20% of the population, but less than half seek medical assistance with the complaint.

**Methods:**

A questionnaire was sent to the 406 GPs listed on the West Sussex Health Authority Medical List to investigate their views of this condition and whether they felt hypnotherapy had a place in its management

**Results:**

38% of general practitioners responded. The achieved sample shared the characteristics of target sample.

Nearly half thought that irritable bowel syndrome (IBS) was a "nervous complaint" and used a combination of "the placebo effect of personal care," therapeutic, and dietary advice. There is considerable divergence in the perceived effectiveness of current approaches. Over 70% thought that hypnotherapy may have a role in the management of patients with IBS; though the majority (68%) felt that this should not be offered by general practitioners. 84% felt that this should be offered by qualified hypnotherapist, with 40% feeling that this should be offered outside the health service.

**Conclusions:**

General practitioners vary in their perceptions of what constitutes effective therapy in IBS. They are willing to consider referral to a qualified hypnotherapist.

## Background

This study explores general practitioners' beliefs about irritable bowel syndrome (IBS), and whether they see hypnotherapy as an appropriate complementary therapy for its management; and if so, who should deliver it.

IBS is estimated to occur in 10–20% of the population in most countries [1]. It is known that less than half of subjects seek medical help for their complaints [2] but it is a common cause for referral to secondary care (referral is advised in patients over 50 years old with changing symptoms) [1].

Over a period of time of between 2 and 5 years, it is thought that there is a 30% turnover of patients having IBS [3]. It therefore uses significant primary care resources over many years.

IBS is typical of many conditions seen in general practice. There is a risk that some of its symptoms may actually represent serious underlying illness and there are a number of conventional medical interventions, but for many patients the interest of their practitioner is paramount [4]. The mainstays of medical management are a high fibre diet and pharmacotherapy [5]. These help to some extent but do not appear to offer cure or permanent remission. There is also little evidence of the effectiveness of dietary advice. Many general practitioners see IBS as a complex bio-psychosocial problem where an appropriate consultation style can be as important as the therapeutic interventions themselves [6].

UK general practitioners are having a greater say in the management of the local health services through the organisation of local health services into Primary Care Trusts (PCT). The latter are responsible for commissioning the health services for their resident population. Via these, general practitioners have the potential to influence the development of services for the treatment of this condition. Most PCTs are financially constrained and cannot meet all the needs of every patient. Services have to be prioritised according to need. The study investigated general practitioners' opinions as to whether hypnotherapy should be provided by Primary Care Trusts, or provided privately.

The possibility of hypnotherapy being used as a primary care level intervention for IBS has developed from the realisation in the 1980's that hypnotherapy was an effective treatment for intractable IBS in a hospital context [7,8]. These trials are good evidence that this therapy is effective in secondary care. The concept of shortening a long-term illness that often requires secondary referral, long-term drug therapy and repeated primary care attendance is an attractive one. No large-scale primary care trial of this sort of treatment has been performed. If a large RCT were to be undertaken it would be important to assess whether the findings would be perceived as relevant and likely to be implemented in a primary care setting.

There is a lack of literature about general practitioners' opinions and few validated questionnaires. A literature search identified two recent studies about IBS in primary care [9,10] which comment on the lack of confidence in the diagnosis of IBS in primary care and the over-dependence on treatment using drugs. An interview study explores medical and lay views of IBS [11]; concluding that patients are affected by medical beliefs about the nature of IBS and suggest that better explanations could be given for the disorder. A questionnaire study of consultants' and GPs' attitudes to functional bowel disorders showed marked differences in the perception of the psychological as opposed to the physical basis for the condition between the two groups – GPs favoured a psychological explanation. However, the study did not investigate alternative therapeutic intervention [12].

There are few studies describing the experiences and attitudes of GPs towards IBS management in primary care, especially pertaining to the use of alternative or psychological treatments. Although it is very likely that treatments such as hypnotherapy and cognitive behaviour therapy would have a prolonged impact on IBS if they were commonly employed at an early stage in the illness, little is known about the attitudes of primary care teams in encouraging this sort of approach.

We therefore conducted this study to discover how general practitioners perceive IBS and if they consider it to be managed effectively by current conventional therapy. We wanted to know if GPs consider hypnotherapy to be an appropriate intervention and whether GPs would refer there patients for it. Finally, we wanted to know whether such a service would be provided within general practice or another setting and if it should be funded by the NHS or be provided from the private sector.

## Methods

### Study setting

A survey was conducted of all general practitioners in West Sussex. The setting for the study is a mixed area. There are market towns and no large cities. Much of the population lives in sub-urban and rural areas. The area has a lower than average deprivation level than the regional norm, but in common with most of the south east of England, income is higher than the national average. As it is a rural area a larger number of practices dispense their own medicines, rather than send patients with a prescription to a local pharmacy [13].

### Questionnaire design and testing

A questionnaire was designed and pre-tested with a pilot group for readability, validity, reliability, acceptability of layout and time taken to complete [14]. The questionnaire is attached as an appendix [see Additional file 1].

The sample was the 406 general practitioners listed as unrestricted principals on the Health Authority's list in 1997. This list included both full and part time general practitioners. The questionnaires were mailed to GPs using the Heath Authority's internal mail system and were returned via the same Health Authority post. At that time, the use of this service was free to the investigator. Once the first group of questionnaires had been returned, the questionnaire was repeated once to those practices that had not responded.

The responses were mostly in the form of a Likert scale. This scale enables measurement of degrees of opinion, so increasing the sensitivity of the analysis. A central category was provided for a neutral response. The design purposefully did not force a polarised choice because it was thought that the treatment would be unlikely to be in common use. It was possible that many general practitioners would genuinely not have an opinion about some of the questions. To prevent acquiescence bias, the questions were worded so that expected responses varied unpredictably according to the direction of the scale [14]. Demographic data were requested, to enable assessment of possibly biased responses due to factors such as age, sex or practice size.

Questionnaire themes included GP perception of IBS, its management, and hypnotherapy as a treatment. It also included questions related to the funding of treatment and the acceptability of hypnotherapy as part of the management of IBS in primary care. The questions were grouped together using these themes. Piloting enabled assessment of face validity and content validity [15]. The constructs being tested were not formally defined, as the purpose of the instrument was to obtain opinion about the subjects of the questions, not to form hypotheses.

### Data analysis

We analysed the data using SPSS (Statistical Package for Social Sciences). The target and achieved samples were compared using the Chi-square test of proportion [16]. Where there is a statistically significant difference between the samples it is included. The results of the main questionnaire are reported as proportions rather than as a score. The purpose of this approach is to ascertain determine general practitioner opinion. Preliminary analysis found that the proportions for the 'strongly agree' and 'strongly disagree' categories are generally small. For clarity of interpretation and reporting, the 5 point-scale was collapsed into 3 where applicable, amalgamating the 'strongly agree' category with the 'agree' category, and 'strongly disagree' with 'disagree'.

## Results

### Response bias

155 (38%) general practitioners of the target sample returned questionnaires after the initial request and one further follow-up reminder.

The characteristics of general practitioners in achieved sample were compared with that of the target sample to assess sample bias. No statistically significant differences were found between the achieved and target samples other than fewer GPs from non-training practices and from single-handed practices responded to the survey. With the exception of these factors, the characteristics of the achieved sample are not very different from that of the population of GPs in West Sussex. A comparison of the achieved and target sample are shown in Table 1. Nine general practitioners (6%) had previously used hypnotherapy.

**Table 1:** Comparison of responders (achieved sample) with all West Sussex GPs (target sample)

**Characteristic**	**Achieved sample**		**Target sample**		**Test**
		
	**%**	**n**	**%**	**n**	
Aged 35 & under	17.4%	27	16.5%	67	
Aged 36–45	43.9%	68	41.6%	169	
Aged 46–55	31.0%	48	28.3%	115	
Aged 56 & over	6.5%	10	12.3%	50	p = 0.17 X^2^
Male	68.4%	106	73.4%	298	
Female	31.0%	48	26.6%	108	p = 0.20 X^2^
Full time	83.9%	130	88.7%	360	
Part time	15.5%	24	11.3%	46	p = 0.09 X^2^
Research active % (95%CI)	12.9% (7.1–18.6)	20	5.7% (3.4–7.9)	23	n.s. Test of Proportion
Training	53.50%	83	16.05%	65	
Non training	43.90%	68	83.95%	341	p < 0.001 X^2^
Single handed	5.2%	8	10.2%	41	
Group	92.3%	143	89.8%	365	p < 0.05 X^2^
**Total**		**155**		**406**	

(Source of GP data in West Sussex: NHS Executive Oct 1997)

### How do general practitioners perceive IBS

45.2% of the respondents agreed with the statement that "IBS is mainly a nervous complaint", and 40% felt that IBS "responds mainly to the placebo effect of personal care and attention". Although many respondents seem to categorise IBS as a 'nervous complaint', conventional medical management with drugs and dietary advice is seen to have a role to play with 45.2% agreeing with the statement that drug therapy works effectively, and 38.7% dietary advice works effectively. A striking finding is that a sizeable minority of GPs were unsure if IBS patients respond to placebo effect (at 37.4%) or to medical therapy (at 41.3%). Combining the 'unsure' and the 'disagree' categories show that the majority of GPs in this survey are uncertain if existing treatment regimes for IBS (drug, dietary advice and placebo effect) are efficacious. These results are set out in Table 2.

**Table 2:** How general practitioners perceive irritable bowel syndrome.

	**Agree**	**Unsure**	**Disagree**	**n=**
Irritable Bowel Syndrome is mainly a 'nervous complaint'	45.2	29.7	23.2	155
Irritable Bowel Syndrome responds mainly to the placebo effect of personal care and attention	40.0	37.4	22.6	155
Drug therapy works effectively in my Irritable Bowel Syndrome patients	45.2	32.9	20.6	155
Dietary advice works effectively in my Irritable Bowel Syndrome patients	38.7	38.7	20.6	155
Irritable Bowel Syndrome responds mainly to medical/therapeutic interventions	39.4	41.3	19.4	155

N.B. the rows do not always sum to 100% as missing responses are included in the analysis but not shown.

### Is care of patients with IBS adequate

Just under half of the respondents (45.2%) agreed that IBS requires more attention in primary care and less than a quarter (22.6%) disagreed with the statement that IBS required more attention. The majority (56.8%) of the respondents felt that it would be possible to manage IBS better in their practices. Only 12.9% disagreed with this statement. The vast majority (84.5%) felt that the present management of IBS is variable with less than 10% of practitioners believing that their care is effective. These results are summarised in Table 3.

**Table 3:** How general practitioners perceive the effectiveness of irritable bowel syndrome management

	**Agree**	**Unsure**	**Disagree**	**n=**
Irritable Bowel Syndrome requires more attention in Primary care	45.2	29.7	22.6	155
It would be practically possible to manage Irritable Bowel Syndrome better in our practice	56.8	28.4	12.9	155
Is your present management of Irritable Bowel Syndrome effective, ineffective or variable?	**Effective**	**Variable**	**Ineffective**	**n=**
	
	9.7	84.5	4.5	155

N.B. the rows do not always sum to 100% as missing responses are included in the analysis but not shown.

### General practitioners views about hypnotherapy and its role in IBS

Three quarters of general practitioners (75.5%) saw hypnotherapy as an alternative therapy. Notwithstanding this, a large majority of general practitioners in this survey agreed that hypnotherapy could help patients who suffer from both physical and psychological problems, 72.9% and 77.4% respectively. However just over a third of general practitioners (34.8%) saw hypnotherapy as potentially dangerous, with just under half feeling unsure (40.6%).

Nearly 84% felt that hypnotherapy was the province of accredited therapists, and only 20% felt that they would be willing to receive training to provide hypnotherapy themselves. The statement that hypnotherapy should be available through an accredited hypnotherapist (83.9% agreed) and not to take this on as a general practitioner (68.4% with 10.3 unsure) were two of the most polarised responses.

If a course of 8 × 30 minutes hypnotherapy were shown to be effective, there would be a willingness amongst most respondents (78.1%) to advise hypnotherapy for some, but definitely not all, IBS patients. 83% of those questioned felt that it was not something that should be offered to every patient with IBS. These results are set out in Table 4.

**Table 4:** What general practitioners think about hypnotherapy and their willingness to refer irritable bowel syndrome patients for hypnotherapy.

	**Agree**	**Unsure**	**Disagree**	**n=**
Is Hypnotherapy an "alternative" not a mainstream therapy?	75.5	17.4	6.5	155
Hypnotherapy could help a sufferer from a physical illness	72.9	22.6	4.5	155
Hypnotherapy could help a sufferer of a Psychological disorder	77.4	18.7	3.9	155
Hypnotherapy could be dangerous	34.8	40.6	24.5	155
Is Hypnotherapy a treatment that you might advise for your patients?	**Yes**	**Neutral**	**No**	**n=**
	38.7	32.9	28.4	155
Hypnotherapy should be available through an accredited Hypnotherapist.	83.9	13.5	1.9	155
Would you be willing to provide Hypnotherapy personally (after training)?	20.6	10.3	68.4	155
**If Hypnotherapy took 8 × 30 minutes sessions to ensure long-lasting remission in Irritable Bowel Syndrome:**	**Yes**		**No**	**n=**
Would this be a cost-effective measure to provide for all Irritable Bowel Syndrome patients?	12.9		83.2	155
Would this be a cost-effective measure to provide for some Irritable Bowel Syndrome patients?	78.1		13.5	155
Would you refer Irritable Bowel Syndrome sufferers to these sessions elsewhere	56.1	28.4	14.8	155

N.B. the rows do not always sum to 100% as missing responses are included in the analysis but not shown.

### How should hypnotherapy for IBS be resourced

There is considerable uncertainty and divergence of opinions amongst the respondents on how hypnotherapy should be funded. Only 36.1% of general practitioners thought that more NHS resources should be used to give IBS sufferers better treatment, yet a slightly larger percentage (44.5%) thought that the money could be better spent elsewhere. The proportion of those answered 'unsure' in response to the above 2 statements are at large at 45.8% and 38.7% respectively.

Whilst just under half (49%) would support their primary care organisation investing in hypnotherapy if it were shown to work, there was less support for providing it within the NHS, at 29%. Most general practitioners (56.8%) thought that hypnotherapy should be provided through private hospitals, with a substantial minority (40%) thinking that insurance companies should pay for it. Details of these responses are shown in Table 5.

**Table 5:** Should hypnotherapy be funded by the National Health Service.

	**Agree**	**Unsure**	**Disagree**	**n=**
National Health Service resources should be used to give Irritable Bowel Syndrome sufferers better treatment	36.1	45.8	18.1	155
National Health Service resources could be better spent on other illnesses	44.5	38.7	16.8	155
I would support my primary care trust investing in Hypnotherapy (if shown to work)	49.0	29.7	21.3	155
Hypnotherapy should be available through the National Health Service	29.0	41.9	28.4	155
Hypnotherapy should be available through private hospitals	56.8	34.2	9.0	155
Medical insurance companies should pay for Hypnotherapy for their clients	40.0	46.5	12.9	155

N.B. the rows do not always sum to 100% as missing responses are included in the analysis but not shown.

Finally despite the divergence and uncertainty on hypnotherapy for IBS sufferers, over three quarters of respondents, 76.3% (table not shown), said that they would be willing to refer patients into a study of hypnotherapy for the treatment of IBS in primary care if it were conducted.

## Discussion

The principal findings of the study are that many general practitioners see IBS as a "nervous condition" to be treated with care and attention, as well as drugs and dietary change. There is considerable divergence in the perception of the effectiveness of current approaches, and a willingness amongst many GPs to refer to qualified hypnotherapists if it can be shown to be effective, even though this treatment is considered "alternative" and potentially dangerous. It is also acknowledged that this could not be made a priority for the NHS, but should be provided privately outside it.

Unfortunately there were not sufficient resources available to perform telephone reminders, and this may have in part accounted for the low response rate. Comparison of the achieved sample with the target sample shows that fewer single-handed practices and more training practices took part in the survey, which may reflect a greater willingness of academically oriented practitioners to participate in research. It is beyond the scope of the present study to estimate how representative the sample is of other areas, especially inner cities and other more deprived areas where private hospital and insurance are simply not an option for most patients.

Existing literature suggests that around 40% of patients in the UK have access to complementary therapy [17]. This study indicated that that only 6% of general practitioners had ever provided hypnotherapy as a treatment, although 38% said that they might advise hypnotherapy under some circumstances.

Other studies show that doctors hold different 'public' and 'private' attitudes to IBS as an illness, and may classify patients informally into 'good' and 'bad' patients [11]. This study seems to show general practitioners responding in a 'public' way, saying that IBS needs more resources and that it could be managed more effectively than it is at present.

Another study concluded that 'Drug usage in the IBS is more than is justified and should, in our view, be minimised [10]. The present study seems to show that just under half of the general practitioners felt that drugs were effective with about a third unsure (whether through pharmacological or placebo effects was not shown).

More research is needed into the spectrum of views of general practitioners about IBS. A qualitative study might have provided more information about general practitioner beliefs about IBS and what they feel should be done to improve services. Additional quantitative work might identify whether there are age-sex differences in practitioners attitudes.

The general practitioners were clearly open to hypnotherapy to help manage IBS. It is unclear whether this was a specific attitude towards hypnotherapy, or a general willingness to promote any psychological or supportive therapy for this illness.

It would also be interesting to investigate the opinions of gastroenterologists or psychosomatic therapists.

## Conclusions

The vast majority of general practitioners think that current management options for IBS are variable in their effectiveness. Many agreed that this condition needs improved treatment options in primary care; and general practitioners seem willing to consider hypnotherapy as one such treatment option if it can be shown to be effective.

## Competing interests

The authors declare that they have no competing interests.

## Authors' contributions

SC conceived the study and developed the questionnaire, TC and S de L analysed the data, S de L with contributions from the other authors wrote the paper.

## Pre-publication history

The pre-publication history for this paper can be accessed here:



## Supplementary Material

Additional File 1 **Appendix 1: The questionnaire **Questionnaire used in the study Click here for file
