# Supplementary material for: General practitioners believe that hypnotherapy could be a useful treatment for irritable bowel syndrome in primary care
Source: BMC Fam Pract. 2004 Oct 13;5:22. doi: 10.1186/1471-2296-5-22 (PMC526280; doi:10.1186/1471-2296-5-22)
Supplement: Additional File 1 — Appendix 1: The questionnaire Questionnaire used in the study [file 1471-2296-5-22-S1.doc]

**Appendix 1: The questionnaire**

**1. Introduction.**

Thank you for your time spent answering these quick questions, it should take less than two minutes to complete. The questionnaire is a research exercise into irritable bowel syndrome, a problem commonly seen in primary care. This is a research interest of mine, and am looking into the feasibility of a larger study to improve its management. Please send the questionnaire back as soon as possible in the attached addressed envelope.

*Thank-you.*

Stephen Cox, General Practitioner.

Dr Rob Morris and Partners in Handcross and Balcombe surgeries.

**Answering the questionnaire:**

Circle your response (once per line only). Some answers are in the following format: Strongly Agree (SA), Agree (A), Unsure/No opinion (U), Disagree (D), Strongly Disagree (SD). There is a space for comments at the end.

**2. An example question:**

Bananas are a very good treatment for Irritable Bowel Syndrome? SA / A / U / D / SD

*The response in this example would signify that you strongly disagree with this statement.*

**3. The questionnaire:**

**a) The need for another treatment in Irritable Bowel Syndrome (Irritable Bowel Syndrome).**

In your opinion:

Irritable Bowel Syndrome requires more attention in Primary care SA / A / U / D / SD

Irritable Bowel Syndrome is mainly a ‘nervous complaint’ SA / A / U / D / SD

Is your present management of Irritable Bowel Syndrome effective,

ineffective or variable? Effec/ Ineff/ Variable

It would be practically possible to manage Irritable Bowel Syndrome better

in our practice SA / A / U / D / SD

Drug therapy works effectively in my Irritable Bowel Syndrome patients

SA / A / U / D / SD

Dietary advice works effectively in my Irritable Bowel Syndrome patients SA / A / U / D / SD

**b) The acceptability of Hypnotherapy as a treatment.**

Have you any personal experience of providing Hypnotherapy? Yes / No

Have you ever received Hypnotherapy? Yes / No

Is Hypnotherapy a treatment that you might advise for your patients? Yes / No / Neutral

Is Hypnotherapy an alternative therapy or a mainstream therapy? Alt / Main / Neutral

Hypnotherapy could help a sufferer from a physical illness SA / A / U / D / SD

Hypnotherapy could help a sufferer of a Psychological disorder SA / A / U / D / SD

Hypnotherapy could be dangerous SA / A / U / D / SD

**c) The expectation that the treatment might work.**

Irritable Bowel Syndrome responds mainly to medical/therapeutic

interventions SA / A / U / D / SD

Irritable Bowel Syndrome responds mainly to the placebo effect of

personal care and attention SA / A / U / D / SD

If Hypnotherapy took eight 30 minute sessions to ensure long-lasting

remission in Irritable Bowel Syndrome, would this be a cost-effective

measure to provide for A) All Irritable Bowel Syndrome patients? Yes / No

B) Some Irritable Bowel Syndrome Patients? Yes / No

**d) The cost and time implications for patient and General Practitioner of Hypnotherapy for Irritable Bowel Syndrome.**

National Health Service resources should be used to give Irritable Bowel

Syndrome sufferers better treatment SA / A / U / D / SD

National Health Service resources could be better spent on other

illnesses SA / A / U / D / SD

I would support my PCG’s investing in Hypnotherapy (if shown to work) SA / A / U / D / SD

Would you be willing to provide Hypnotherapy personally (after training)? Yes / No / Neutral

Would you refer Irritable Bowel Syndrome sufferers to these sessions

elsewhere Yes / No / Neutral

Hypnotherapy should be available through the National Health Service SA / A / U / D / SD

Hypnotherapy should be available through private hospitals SA / A / U / D / SD

Hypnotherapy should be available through an accredited Hypnotherapist SA / A / U / D / SD

Medical insurance companies should pay for Hypnotherapy for their clients SA / A / U / D / SD

**e) The possibility of taking part in the real trial.**

In principle would you refer Irritable Bowel Syndrome sufferers

to a trial of therapy? Yes / No

Do you know of any other local trials in this subject area? Yes / No

**f) Demographic details**

Is your practice a) Single Handed or a Group

b) <5000 /5-10,000 /10-20,000/>20,000 patients

c) Training /Non-training

d) STaRNet practice/ MRC General Practitioner Research Framework practice/ Non-research

e) Rural/ Urban/ Mixed/ Neither

Are you a) 25-35/ 36-45/ 46-55/ 56-65+

b) Male/ female

c) Full/ Part time

**4. Please write any comments you may wish to make in this space.**

*(Continue on another sheet if needed)*

**Many thanks for your time and patience.**

Please send this questionnaire back to W. Sussex Health Authority in the normal internal mail. An addressed envelope is provided. The Health Authority, who have kindly agreed to act as a post office for me, will NOT see your answers.

**6. Do you want a summary of the findings?**

A summary of the findings from this questionnaire will be sent to you if you stamp this form.

Stamp here, for a copy of the results:
